# Serum biomarkers in primary mitochondrial disorders

**DOI:** 10.1093/braincomms/fcaa222

**Published:** 2021-01-04

**Authors:** Kristin N Varhaug, Omar Hikmat, Hanne Linda Nakkestad, Christian A Vedeler, Laurence A Bindoff

**Affiliations:** 1 Department of Neurology, Haukeland University Hospital, Bergen, Norway; 2 Department of Clinical Medicine, University of Bergen, Bergen, Norway; 3 Department of Paediatrics and Adolescents, Haukeland University Hospital, Bergen, Norway; 4 Department of Neurology, Neuro-SysMed, Haukeland University Hospital, Bergen, Norway

**Keywords:** mitochondrial disease, neurofilaments, FGF-21, GDF-15

## Abstract

The aim of this study was to explore the utility of the serum biomarkers neurofilament light chain, fibroblast growth factor 21 and growth and differentiation factor 15 in diagnosing primary mitochondrial disorders. We measured serum neurofilament light chain, fibroblast growth factor 21 and growth and differentiation factor 15 in 26 patients with a genetically proven mitochondrial disease. Fibroblast growth factor 21 and growth and differentiation factor 15 were measured by enzyme-linked immunosorbent assay and neurofilament light chain with the Simoa assay. Neurofilament light chain was highest in patients with multi-systemic involvement that included the central nervous system such as those with the m.3242A>G mutation. Mean neurofilament light chain was also highest in patients with epilepsy versus those without [49.74 pg/ml versus 19.7 pg/ml (*P* = 0.015)], whereas fibroblast growth factor 21 and growth and differentiation factor 15 levels were highest in patients with prominent myopathy, such as those with single-mitochondrial DNA deletion. Our results suggest that the combination of neurofilament light chain, fibroblast growth factor 21 and growth and differentiation factor 15 is useful in the diagnostic evaluation of mitochondrial disease. Growth and differentiation factor 15 and fibroblast growth factor 21 identify those with muscle involvement, whereas neurofilament light chain is a clear marker for central nervous system involvement independent of underlying mitochondrial pathology. Levels of neurofilament light chain appear to correlate with the degree of ongoing damage suggesting, therefore, that monitoring neurofilament light chain levels may provide prognostic information and a way of monitoring disease activity.

## Teaser

Varhaug *et al.* compared the utility of three serum biomarkers in mitochondrial disease—neurofilament light chain (NF-L), fibroblast growth factor-21 (FGF-21) and growth differentiation factor 15 (GDF-15). As expected, FGF-21 and GDF-15 were highest in patients with predominantly muscular symptoms. NF-L correlated with structural involvement of the brain especially where epilepsy occurred.

## Graphical Abstract

**Graphical Abstract:**
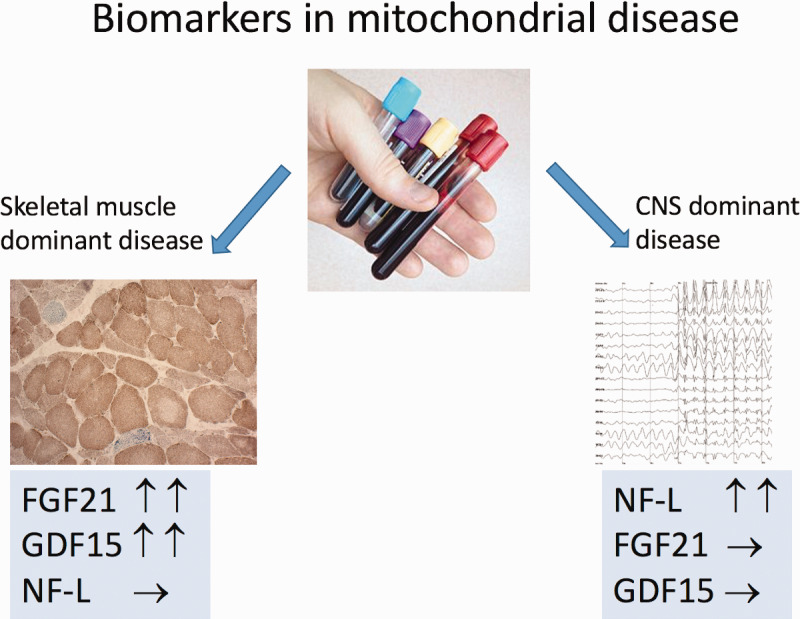

## Introduction

Mitochondrial diseases are a heterogeneous group of disorders arising from mutations in genes encoded either in the nucleus or in the mitochondrion’s own multi-copy genome (mitochondrial DNA, mtDNA).

Diagnosing mitochondrial disease is challenging because of both the clinical heterogeneity and the paucity of clinically useful biomarkers. Elevated blood lactate is an inconsistent finding and diagnosis has relied heavily on muscle biopsy (Turnbull, 2011). Two new biomarkers, fibroblast growth factor 21 (FGF-21) and growth and differentiation factor 15 (GDF-15), have been described for mitochondrial disease. FGF-21 is a hormone-like cytokine secreted in response to starvation and leads to mobilization of lipid stores and production of ketone bodies (Tyynismaa *et al.*, 2010). GDF-15 is a member of the transforming growth factor beta superfamily (Weiss and Attisano, 2013), and has a role in regulating cellular response to stress and inflammation (Boenzi and Diodato, 2018). FGF-21 and GDF-15 are both primarily elevated in mitochondrial disease affecting muscle (Suomalainen *et al.*, 2011; Kalko *et al.*, 2014) although GDF-15 appears more sensitive in detecting mitochondrial dysfunction affecting other organs (Davis *et al.*, 2016).

Neurofilament light-chain (NF-L) is a neuron-specific protein. NF-L is a marker of disease activity and progression has been evaluated in a number of different neurological conditions (Varhaug *et al.*, 2019). Increased NF-L levels reflect ongoing neuronal damage, irrespective of the underlying pathology, making it a potentially interesting biomarker also in mitochondrial disorders.

Our aim was to measure these non-invasive and easily obtained serum biomarkers in a cohort of mitochondrial patients, and to evaluate diagnostic and clinical usefulness.

## Materials and methods

### Study design and patients

Twenty-six patients with a genetically confirmed mitochondrial disorder were recruited at the outpatient clinic, Department of Neurology, Haukeland University Hospital, Bergen, Norway.

### Serum sampling and analysis

Serum samples were stored at −80°C until analysis.

The concentration of NF-L was determined using the Simoa assay (UmanDiagnostics, Quanterix). The concentrations of FGF-21 and GDF-15 were measured in duplicate samples by ELISA according to the manufacturer’s protocol (BioVendor, Brno, Czech Republic).

### Statistical analyses

The data were processed using SPSS v25 (IBM). Statistical differences were determined using Kruskal–Wallis and Mann–Whitney *U* non-parametric tests, with Benjamini–Hochberg adjustment. A *P*-value of <0.05 was considered statistically significant. Correlations were assessed with Spearman’s correlation test. Graphs were made using Graphpad prism 8.

### Ethical approval

The study was approved by the Norwegian Regional Committee for Medical and Health Research Ethics (No: 2019/479), and written consent was obtained from all patients.

### Missing data

FGF-21 measurement was missing from one patient.

### Data availability

The data that supports the findings of this study is available from the corresponding author upon reasonable request.

## Results

### Clinical features

Demographic characteristics are summarized in Table 1. All patients were adults, apart from one, aged 14 years. The mean age was 48.2 years [standard deviation (SD), 17.8].

**Table 1:** Characteristics of the patients

	MtDNA point mutations	*N*	Single deletions	Nuclear gene mutations	*N*
Genetic diagnose	*8344 A>G*	2		*POLG*	7
	*3243 A>G*	4		*TWINKL*	2
	*13271 T>C*	1		*PITRM1*	1
	*5556 G>C*	1		*DHX30*	1
				*ICSU*	1
Age (mean years)	58		43	47	
Gender (female %)	63 %		100 %	83 %	
Total		8	6		12

#### NF-L

**Figure 1:**
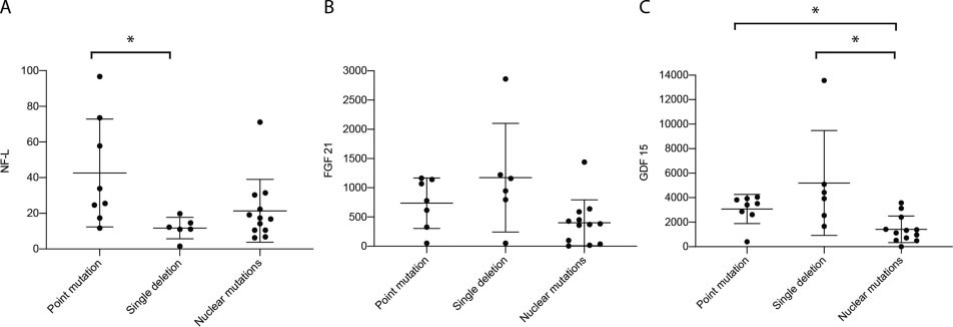
**Biomarkers when sub-classified by genetic diagnosis.** The differences in mean of the three biomarkers when the patients are genetically classified in point mutations, single deletions and nuclear mutations are shown. All values are expressed in pg/ml. The bars represent mean and standard deviation in NF-L (**A**), FGF-21 (**B**) and GDF-15 (**C**). There were significant differences between point mutations and single deletions in NF-L, and nuclear mutations and both point mutations and single deletions in GDF-15. Significant differences are marked with asterisk (*).

The overall mean concentration of serum NF-L was 25.70 pg/ml (SD = 23.4 pg/ml). When sub-classifying by genetic diagnosis, mean NF-L concentration was 43.89 pg/ml (SD = 32.4 pg/ml) in patients with mtDNA point mutations, 11.70 pg/ml (SD = 5.6 pg/ml) in mtDNA single deletions and 21.39 pg/ml (SD = 17.6 pg/ml) in nuclear gene mutations (Fig. 1A). The difference in NF-L levels between mtDNA point mutations and single deletions was significant (*P* = 0.024). There was a trend towards significant difference between mtDNA point mutations and nuclear mutations (*P* = 0.0585).

**Figure 2:**
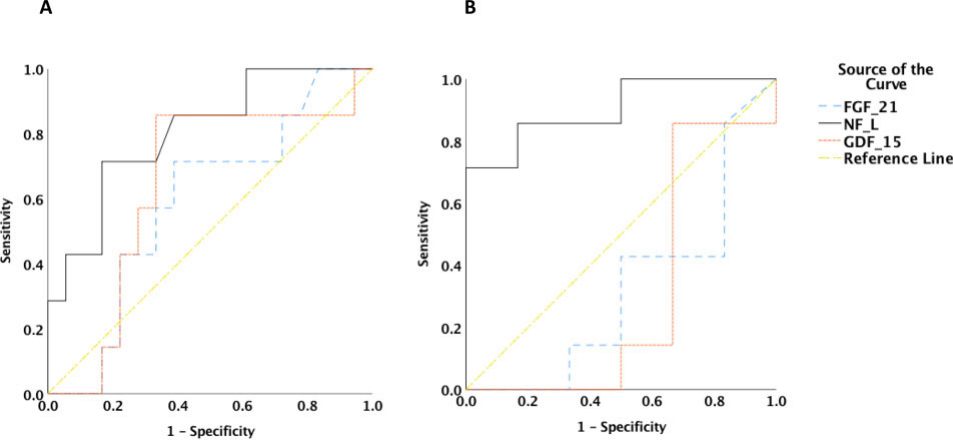
**ROC curves for the three biomarkers. Receiver-operating characteristics curves for the three biomarkers in patients.** (**A**) Point mutations versus single deletions and nuclear mutations; AUC = 0.806, (*P* = 0.02). (**B**) Point mutations versus single deletions; AUC = 0.905 (*P* = 0.015).

NF-L could differentiate between patients with mtDNA point mutations and the other two groups with an Area under the curve (AUC) of 0.806 (Fig. 2A). When compared to single deletions only, the AUC was 0.905 (Fig. 2B). We also looked at patients with epilepsy regardless of the genetic aetiology. The mean NF-L level in the epilepsy group (five patients) was 49.74 pg/ml versus 19.7 pg/ml in the non-epilepsy group (21 patients) (*P* = 0.015).

In two patients (with POLG disease and m.3243A>G-mutation), we had one blood sample taken at an earlier time point and could calculate the annual increase in the concentrations of NF-L. Normal annual increase has been previously estimated to be about 2.2%/year (Disanto *et al.*, 2017). The annual increase for the POLG-patient was 4.24%, the m.3243A>G patient 7.4%.

### FGF-21 and GDF-15

The group mean concentration of FGF-21 was 679.77 pg/ml (SD = 630.07 pg/ml) and GDF-15 was 2802.43 (SD = 2628.04 pg/ml). In mtDNA point mutations, mean FGF-21 concentration was 735.10 pg/ml (SD = 430.2 pg/ml), in single deletions 1172.53 pg/ml (range: SD = 927.8 pg/ml) and in nuclear mutations 401.1 pg/ml (SD = 393.5 pg/ml) (Fig. 1B). There were no significant differences between the FGF-21 levels and the different underlying genetic groups (*P* = 0.052). The sub-group mean for GDF-15 concentration in mtDNA point mutations was 3027.27 pg/ml (SD = 1278.0 pg/ml), 5202.59 pg/ml (SD = 4281.3 pg/ml) in single deletions and 1420.54 pg/ml (SD = 1087.7 pg/ml) in nuclear mutations (Fig. 1C). The difference in GDF-15 levels between nuclear mutations and point mutations and single deletions was significant (*P* = 0.024 and *P* = 0.006, respectively).

**Figure 3:**
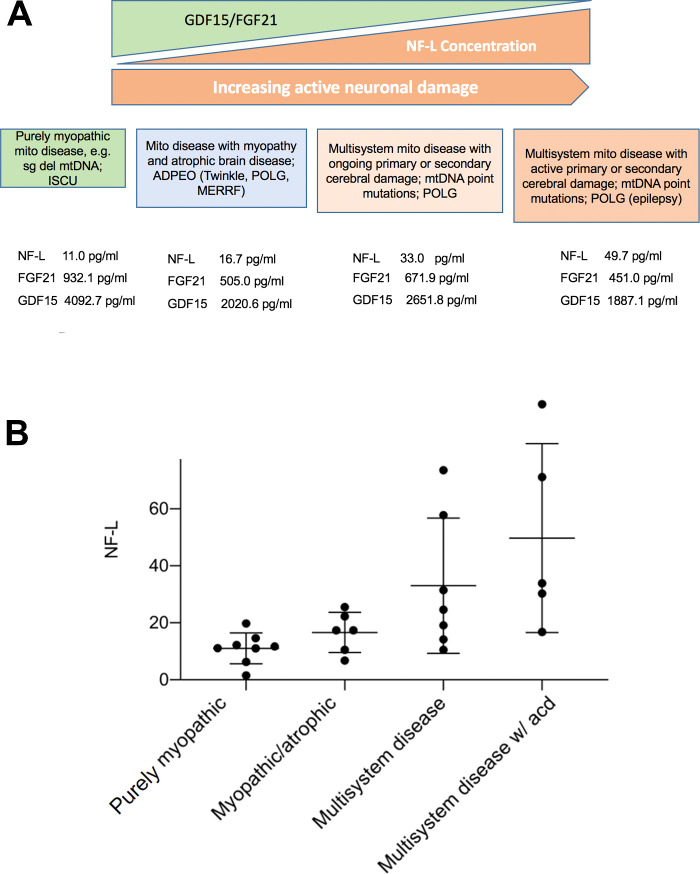
**The biomarkers ability to differentiate clinical groups.** There is a reciprocal trend in NF-L to FGF-21 and GDF-15, here illustrated by sub-classifying the patients by degree of CNS involvement. Brain involvement was sub-divided into slowly progressive atrophy, neurodegenerative disease with ongoing neuronal loss and conditions in which there would be more acute neuronal loss due to ongoing seizures or ischaemic damage (acd, active cerebral damage). The differences are most pronounced when looking at NF-L. (**B**) The differences between the clinical groups defined in (**A**) are significant (*P* = 0.012).

There was a significant correlation between FGF-21 and GDF-15 (*r *=* *0.671, *P* < 0.001, Spearman’s correlation). There was no correlation between FGF-21 or GDF-15 and NF-L. However, NF-L showed a reciprocal trend to FGF-21 and GDF-15 when the patients were divided in clinical groups, from pure myopathic to multi-system disease with active cerebral damage (Fig. 3A). The differences in NF-L were significant (*P* = 0.012) (Fig. 3B).

## Discussion

Our study shows that NF-L provides important clinical information about the presence of active neuronal damage in mitochondrial disease. We also confirmed that FGF-21 and GDF-15 are useful biomarkers for mitochondrial disease in which myopathy is the major or only manifestation (Lehtonen *et al.*, 2016; Montero *et al.*, 2016). Intriguingly, NF-L showed a reciprocal trend to FGF-21 and GDF-15, suggesting that these three biomarkers may complement each other as diagnostic tools in the investigation of mitochondrial disorders.

The overall mean NF-L concentration in patients with mitochondrial disease was 25.70 pg/ml. This level is pathological (Varhaug *et al.*, 2019). When we classified our study cohort according to the presence and type of central nervous system (CNS) involvement, we observed clear differences in the concentrations of NF-L (Fig. 3). In multi-systemic disorders with CNS involvement, particularly those associated with point mutations such as the m.3243A>G-mutation, the highest levels of NF-L were observed. In contrast, in patients with single deletions, and a pure (or almost pure) myopathic phenotype, the levels of NF-L were normal.

We found significant differences in NF-L levels according to the type of CNS involvement. First, those with epilepsy had much higher NF-L levels than those without. This is of major importance since epilepsy is one of the most important prognostic factors, especially in POLG disease, and swift detection and treatment of any epileptic seizure activity is vital (Engelsen *et al.*, 2008). The stroke-like episodes which are often seen in both POLG disease and MELAS caused by m.3243A>G are medical emergencies (Ng *et al.*, 2019), and an elevated NF-L at time of diagnosis, or one that shows a rising profile, may indicate a higher risk of seizures or stroke-like episodes. We suggest, therefore, that patients with mutations known to cause severe neurodegenerative damage, such as *POLG* and m.3243A>G-mutations, should have serum NF-L levels monitored regularly (e.g. every 3 months). Rising levels should trigger further investigations including MRI, EEG and consideration of whether anti-convulsant treatment should be initiated even prior to a first seizure event.

We also found differences in the levels of NF-L between patients in whom the CNS process was slowly progressive, i.e. only atrophy, compared to those in which the process was more destructive, particularly those with ongoing epilepsy. This is exemplified by looking at patients with POLG disease: those with ataxia and no epilepsy had lower levels than those who had already developed epilepsy.

A previous study in paediatric patients found that NF-L could be used as a marker for mitochondrial encephalopathies, and that it is correlated with MRI abnormalities and poor prognostic outcome (Sofou *et al.*, 2019). They also noted a marked high NF-L level in patients with MELAS. This study was, however, performed in cerebrospinal fluid and we feel that blood is a more accessible tissue and one that will better facilitate repeated measurements.

We believe that, as in other disease such as multiple sclerosis (Varhaug *et al.*, 2018, 2019), the most important use of NF-L in mitochondrial disease will be the patient’s own variation. For example, in a patient with m.3243A>G-mutation there was a marked and pathological elevation of 7.4%. This reflects what we saw when comparing disease sub-groups; those with destructive mutations such as the m.3243A>G-mutations had pathological NF-L levels that continued to rise. Interestingly, a study looking at plasma levels of cell-free mtDNA in mitochondrial patients (Maresca *et al.*, 2020) showed that these were higher in patients with MELAS than patients with mitochondrial disease due to nuclear gene mutations. They also found that levels increased over time in patients with MELAS, suggesting that cell-free mtDNA may too function as a marker of disease progression.

Previous studies have identified a threshold value of 350.0 pg/ml for FGF-21 in mitochondrial disease (Yatsuga *et al.*, 2015). The threshold for GDF-15 depends on the kit used and with the BioVendor kit, used in this study, the threshold has been set to 2330 pg/ml (Davis *et al.*, 2016). We found that patients in our sub-group of nuclear mutations had a mean GDF-15 level lower than threshold. Since GDF-15 is considered as a more sensitive marker for mitochondrial disorder, in general, and not just in mitochondrial muscle involvement (Davis *et al.*, 2016), this was surprising. The majority of our patients with nuclear gene mutations were adults with *POLG* mutations and phenotypes consisting of epilepsy, neuropathy and/or ataxia. Myopathy was not a major feature, and the low levels of GDF-15 could therefore be a reflection of this. Thus, while GDF-15 might, under certain conditions be a broader biomarker, it remains essentially a mitochondrial myopathy marker. That myopathy was not a major feature was supported by our FGF-21 results: FGF-21 levels were also lower in the group of patients with nuclear gene mutations than it could be expected, but similar to those reported earlier in ataxic POLG patients (Suomalainen *et al.*, 2011).

Both FGF-21 and GDF-15 were significantly elevated in patients with single mtDNA deletion, and high in those with a significant degree of muscle involvement. Correlation between these two biomarkers was also good, confirming the results of earlier studies (Montero *et al.*, 2016). Our impression is that these biomarkers are good when mitochondrial myopathy is present, but less specific when muscle is not involved. This is in accordance with the previous findings, showing positive correlation between FGF-21 and GDF-15, as well as with lactic acid and creatine (Maresca *et al.*, 2020). In contrast, NF-L appears much more sensitive at detecting the presence of CNS damage. We find significant differences in the levels of NF-L between the three genetic sub-groups, and the levels of AUC suggest that NF-L could be a useful tool in identifying the mitochondrial patients with more complex cerebral involvement. In combination with FGF-21 and GDF-15, NF-L, and potentially cell-free mtDNA, may contribute to narrowing the choice of diagnostic test in cases of suspected mitochondrial disease.

Mitochondrial disorders are rare and heterogenous disorders making large studies difficult. However, prospective multi-centre studies will be necessary to confirm the role of NF-L as a biomarker, and how it, together with GDF-15 and FGF-21, can be used most profitably in the investigation of mitochondrial disease. We would, however, like to suggest that NF-L be added to the list of biomarkers that should be monitored during clinical trials in mitochondrial disease (Mancuso *et al.*, 2017).

## Conclusion

In conclusion, our study indicates that we now have three novel serum biomarkers for use in mitochondrial disorders. The combination of these biomarkers can be used both as diagnostic tools and in clinical follow-up. We believe that there is also an important role for these biomarkers in future natural history studies and drug trials.

## Abbreviations

AUC =: Area under the curve
FGF-21 =: fibroblast growth factor 21
GDF-15 =: growth and differentiation factor 15
MELAS =: mitochondrial encephalomyopathy, lactic acidosis and stroke-like episodes
mtDNA =: mitochondrial DNA
NF-L =: neurofilament light chain
POLG =: polymerase gamma
SD =: standard deviation
